# Impact of vaccine mandates and removals on COVID-19 vaccine uptake in Australia and international comparators: a study protocol

**DOI:** 10.1136/bmjopen-2024-097412

**Published:** 2025-07-07

**Authors:** Aregawi Gebremedhin Gebremariam, Mesfin Genie, Huong Le, Katie Attwell, Bette Liu, Annette K Regan, Frank H Beard, Kristine Macartney, Francesco Paolucci, Hannah Catherine Moore, Christopher C Blyth

**Affiliations:** 1 Newcastle Business School, The University of Newcastle Australia, Newcastle, New South Wales, Australia; 2 Department of Public Health, Policy and Systems, University of Liverpool, Liverpool, UK; 3 Health Economics Research Unit, University of Aberdeen, Aberdeen, UK; 4 Wesfarmers Centre of Vaccines and Infectious Diseases, The Kids Research Institute Australia, Nedlands, Western Australia, Australia; 5 Centre for Child Health Research, The University of Western Australia, Perth, Western Australia, Australia; 6 Curtin University School of Population Health, Perth, Western Australia, Australia; 7 School of Social Sciences, The University of Western Australia, Perth, Western Australia, Australia; 8 School of Public Health and Community Medicine, University of New South Wales, Sydney, New South Wales, Australia; 9 Department of Research and Evaluation, Kaiser Permanente Southern California, Pasadena, CA, USA; 10 National Centre for Immunisation Research and Surveillance, Westmead, New South Wales, Australia; 11 The University of Sydney School of Public Health, Sydney, New South Wales, Australia; 12 National Centre for Immunisation Research and Surveillance of Vaccine Preventable Diseases, Westmead, New South Wales, Australia; 13 The University of Sydney Children’s Hospital Westmead Clinical School, Westmead, New South Wales, Australia; 14 School of Medicine and Pharmacology, The University of Western Australia, Perth, Western Australia, Australia; 15 Department of Infectious Diseases, Perth Children’s Hospital, Perth, Western Australia, Australia; 16 Department of Microbiology, PathWest Laboratory Medicine WA, Nedlands, Western Australia, Australia

**Keywords:** COVID-19, Health policy, Vaccination

## Abstract

**Abstract:**

**Background:**

Vaccination against SARS-CoV-2 was a crucial public health measure during the COVID-19 pandemic. Among the multiple strategies developed to increase vaccine uptake, governments often employed vaccine mandates. However, little evidence exists globally about the impact of these mandates and their subsequent removal on vaccine uptake, including in Australia, France, Italy and the USA. The aim of this study is to provide a protocol to evaluate and quantify the impact of COVID-19 vaccine mandates and removals on vaccine uptake in these countries, with a specific focus on comparing Australian policies with those from Europe and the USA. Actualising the work outlined in this protocol will help to provide policy and technical guidance for future pandemic preparedness and routine immunisation programmes.

**Methods and analysis:**

This protocol outlines a retrospective study using existing data sources including Australian Immunisation Register-Person Level Integrated Data Asset for Australia and publicly available data for France, Italy and California (USA). Causal inference methods such as interrupted time series, regression discontinuity design, difference-in-differences, matching and synthetic control will be employed to assess the estimated effects of vaccine mandates and removals on vaccine uptake.

**Ethics and dissemination:**

The University of Newcastle’s human research ethics committee has approved the study (reference number: H-2024-0160). Peer-reviewed papers will be submitted, and results will be presented at public health, immunisation and health economic conferences nationally and internationally. A lay summary will be published on the MandEval website.

**STRENGTHS AND LIMITATIONS OF THIS STUDY**

- The study leverages high-quality and detailed data sources, such as the Australian Immunisation Register-Person Level Integrated Data Asset, enabling robust and granular analysis of vaccine uptake across multiple dimensions, including demographics and occupational categories.
- By incorporating data from Australia, the USA, France and Italy, the study provides an international comparative perspective, increasing the generalisability and relevance of findings to various policy contexts.
- The use of diverse causal inference techniques (eg, interrupted time series, difference-in-differences, synthetic control methods) ensures rigorous analysis of policy impacts and accommodates variations in mandate implementation and context.
- The careful delineation of mandate timelines (announcement, implementation and removal) and integration of contextual factors (eg, socioeconomic and epidemiological variables) enhance the precision and credibility of the policy impact assessment.
- The reliance on retrospective and pre-existing data might introduce limitations related to data completeness, accuracy or biases.

## Introduction

 COVID-19 vaccine mandates introduced by Australian state and territory governments and in multiple other countries consisted of employment mandates, allowing only vaccinated persons to work in their usual roles and public space mandates, requiring vaccinations to access events, entertainment and hospitality venues, etc.^1^ Governments announced, implemented and removed these mandates at different time points. They introduced employment mandates progressively, expanding to cover different occupations. Public space mandates covered different environments, and though most states implemented them, elements of their design and scope varied.

Governments worldwide implemented mandates to ensure high uptake of COVID-19 vaccines, but other goals lay behind this, including the protection of vulnerable individuals, the prevention of absenteeism in critical workforces and reducing stress on healthcare systems. Each of these goals targets different subpopulations and has its own set of considerations and outcomes, yet these have not been thoroughly examined. Although governments introducing these policies sought high levels of vaccine coverage across different occupations and population groups, the impact of vaccine mandates on uptake is unclear, particularly among occupational and other demographic subgroups, and little is known about how mandate removal influenced uptake of subsequent doses. Moreover, the limited evidence base evaluating the impact of mandates on vaccine uptake presents challenges for policymakers to introduce similar policies in response to future public health emergencies or to support routine vaccine programmes. Given the lack of evidence in terms of the extent to which mandates’ implementation and removal affect vaccine uptake, it is timely to explore the impact of such vaccination policies on vaccine uptake.

Existing investigations into COVID-19 vaccine mandates have uncovered mixed outcomes in terms of vaccine uptake, which are likely to be influenced by regional and contextual factors. Studies show that COVID-19 vaccine mandates appeared to increase vaccine uptake, although with outcomes varying significantly based on regional and demographic contexts (see table 1). These studies highlight that while mandates generally boost vaccination rates, the magnitude and sustainability of these effects are likely to be contingent on specific policy designs, the timing of implementation and the demographic segments targeted.

**Table 1:** Summary of existing literature on vaccine mandates and uptake

Study	Population	Type of mandate	Analytical approach	Findings
Karaivanov *et al*^22^	10 Canadian states (15 June to 14 September 2021) and France, Italy and Germany (15 June to 31 December 2021)	Government-mandated proof of COVID-19 vaccination requirements for access to public venues and non-essential businesses	Differences-in-Differences and interrupted time series analysis	Vaccine mandates were found to improve vaccine uptake, vaccination rate increases by about 5 percentage points (p.p.) for Canadian provinces, 8 p.p. for France, 12 p.p. for Italy and 4.7 p.p. for Germany.
Mills and Rüttenauer^23^	Six countries - Denmark, Israel, Italy, France, Germany and Switzerland (March to November 2021 - including for 19 control countries)	Mandatory COVID-19 certification or certification requirements	Synthetic control method	Mandates increased vaccine uptake, particularly among younger populations and when access restrictions were linked to certain settings, such as nightclubs and large events.
Fitzpatrick *et al*^24^	Canadian provinces (June to November 2021)	Proof-of-vaccination mandates announcement	Event study regression analysis	Mandates led to a significant, although temporary, increase in first-dose uptake, 17.5% increase over the number of vaccinations, especially among individuals younger than fifty years.
Maquiling *et al*^25^	Canadian provinces (July to November 2021)	COVID-19 vaccine mandate announcements	Interrupted time series analysis with a quasibinomial autoregressive model	Increases in vaccine uptake following mandate announcements in several provinces (about 4%–8% increase in vaccination coverage over 10 weeks).

## Aims

Building on this prior research, this present study aims to understand the impact of the announcement of vaccine mandates and their subsequent removals on vaccine uptake, both at the population level and-where possible-within key demographic subgroups, including those targeted by the policy or otherwise relevant for demographic reasons (eg, ethnicity/cultural background, sex and class). By exploring retrospective data, we aim primarily to evaluate the impact of COVID-19 vaccine mandates and removals on vaccine uptake in Australia as the coordinating study site, where most of our study team is based and where we have access to a unique linked dataset. This study will also investigate comparators in France, Italy and California (USA), all of which implemented public space and employment mandates similar to those used in Australia. International comparators have been chosen specifically: Italy was the first European country to mandate COVID-19 vaccination (in healthcare workers); France and Italy both experimented with a range of mandate designs; and of all American states, California introduced the broadest mandates. The primary outcome is the uptake of any vaccine dose, with secondary outcomes including completion of the primary vaccination series and receipt of booster doses, where data allow.

Specifically, this study will:

- Quantify the effect of vaccine mandates (announcement and removal) on vaccine uptake at a population and subpopulation level.
- Investigate the specific effects of distinct mandate designs and enforcement (eg, employment and public space mandates) on COVID-19 vaccine uptake, considering variables such as sex, age and geographical location.

The results from this study could potentially contribute to the evidence base on the effects of vaccine mandates and their removals currently missing from the Australian and global health policy landscape, helping future policymakers make decisions informed by whether, how and what makes mandates effective for increasing vaccine uptake. Furthermore, it is important to provide evidence/guidance for future studies on further mandate experiences.

## Methods and analysis

### Overview of approaches and methods

#### Data sources

This study is part of a broader project, running from January 2024 to December 2027, that aims to evaluate the impacts of COVID-19 vaccine mandates. The study will primarily use novel, nationally linked individual-level population-based data sets. In the Australian context, we will use the Australian Immunisation Register (AIR) linked with the 2021 Census and a wealth of other administrative data in the Person Level Integrated Data Asset (PLIDA) produced from the Australian Government’s Multi-Agency Data Integration Project. PLIDA is a secure data asset combining information on health, education, government payments, income and taxation, employment, and population demographics (including the Census) over time.^2^ PLIDA data support government decision-making and academic research. The Australian Bureau of Statistics (ABS) is the Accredited Integrating Authority for PLIDA, collecting and combining datasets and providing access to authorised researchers. PLIDA includes administrative data shared with the ABS and ABS survey data. PLIDA evolves to include new and updated sources of data. The ABS only links data to PLIDA that are reasonably necessary for an approved purpose.^3^ This unique Australian data source allows us to evaluate the impact of Australia’s employment mandates by profession (eg, teachers) and assess uptake by various subgroups at times of mandate announcement/post removal. Based on known announcement and implementation dates of vaccine mandates and removals, we can use this resource to quantitatively assess their impacts on vaccine uptake.

The AIR^4^ is the national immunisation register in Australia, including a record of all COVID-19 vaccines in Australia reported as administered by immunisation providers. Reporting of COVID-19, influenza, Japanese encephalitis virus vaccines, and all other vaccines listed on the national immunisation programme is mandatory. The AIR records the brand, dose and date of vaccination given to a specific individual. Coverage on this register includes all vaccine records given to all people in Australia. This includes all people on Medicare (though people have the option to opt out, this group represents a small minority), as well as people not entitled to Medicare but who are immunised in Australia by a registered immunisation provider. AIR also includes a COVID-19 immunisation record for people immunised overseas who have transferred their record to Australia. AIR data in PLIDA is currently updated weekly, and for this study, we will include vaccines administered up to 31 December 2023. We will also use the 2021 Census of Population and Housing, the scope of which was all usual residents of Australia on the night of 9 August 2021 living in private and non-private dwellings; efforts are also made to reach street-present people. Census data include comprehensive socioeconomic, demographic and cultural characteristics of Australians.^5^

The novelty of AIR-PLIDA data in Australia also allows us to evaluate the impact of employment mandates on vaccine uptake in people whose tax returns and/or census data show that they work in specific professions (eg, teachers). We will compile and use announcement and implementation dates of vaccine mandates in addition to context-specific information (epidemiological situations, external factors, etc) from various sources, including state government websites and major news websites. Further, we will use government sources to collect information occurring at different times, such as epidemiological situations (number of infections, hospitalisations and deaths), external factors (outbreaks, border closures and lockdowns, access barriers) and state and country-level information such as internal border closures, lockdowns, travel regulations, vaccine supply, elections, etc. Attending to these factors will help us to assess time and location-specific factors that may have also contributed to the uptake of vaccines during the time periods under question. While we do not yet have access to national notifiable diseases data or hospitalisation data linked to AIR-PLIDA, and current mortality data within PLIDA extends only to 2021, we anticipate requesting updated death records as they become accessible. We will also use socioeconomic indexes for areas (SEIFAa product developed by the ABS that ranks areas in Australia according to relative socioeconomic advantage and disadvantage^6^), remoteness index,^7^ citizenship status, country of birth, year of arrival in Australia, religious affiliations and additional socioeconomic variables in the census, where feasible/possible, to consider how mandates impact different segments of the Australian population in variable ways. Within the ABS data laboratory, we will leverage AIR-PLIDA to generate studies focused on specific groups (eg, by occupation such as healthcare, aged care, educators/teachers or service sector employees or by age groups such as children or the elderly).

Data sources for the California segment of the study will be derived from aggregated state and county-level metrics. State and county-level weekly/daily vaccination figures will be used as our primary outcome variable. Data on the proportion of the population that has received one dose, two doses and booster doses of a COVID-19 vaccine will be retrieved from Our World in Data^8^ and official state platforms. Detailed statistics on vaccine administration, testing, hospitalisations and deaths, broken down by county and further stratified by race, ethnicity and age groups, will be collated from the California Department of Public Health’s website.^9^

As we will be using country-level data for France and Italy, we will use country-level weekly vaccine doses as our outcome variable of interest. For both Italy and France, we will retrieve data from various sources, including ‘Our World in Data’^8^ and the European Centre for Disease Prevention and Control (ECDC) and The European Surveillance System (TESSy).^10^ The TESSy dataset has data in a common format between Italy and France and relies on the national data.

For France, the share of the population who received one dose of a COVID-19 vaccine, two doses of a COVID-19 vaccine and booster doses will be retrieved from ‘Our World in Data’,^8^ from the official government sources^11^ and the data collected by Santé Publique France (SPF). Additionally, the French state health insurance service (Assurance Maladie) provides public datasets of vaccination rates in France. These datasets are based on aggregated individual data on beneficiaries of the national health insurance service who received healthcare in the past year. The vaccination data are available by age group: 00–19 years, 20–39 years, 40–54 years, 55–64 years, 65–74 years, 75 years and over.

For Italy, the share of the population who received one dose of a COVID-19 vaccine, two doses of a COVID-19 vaccine and booster doses will be obtained from ‘Our World in Data’.^8^ Additionally, we will access data from the Italian Ministry of Health, which maintains a publicly available repository in open format, containing comprehensive data on the delivery and administration of COVID-19 vaccines across different regions of Italy.^12^ This repository includes various datasets, including details on vaccine deliveries categorised by date and regions; total daily deliveries of vaccines by region; total deliveries and administrations to date (including the percentage of doses administered in relation to the total delivered) by region; daily vaccine administrations categorised by region and age group of vaccinated individuals; information on the population eligible for additional/booster doses and second booster doses; and data on population aged 60 and above who have received a vaccine dose categorised by region and age ranges.

#### Outcome measures

We will assess three primary endpoints: (1) receipt of at least one dose of COVID-19 vaccine, (2) completion of the primary vaccination series (two COVID-19 vaccine doses) and (3) receipt of a COVID-19 booster dose. We will take into account that the Johnson & Johnson vaccine, which was administered in the USA and Italy in limited quantities, required only a single dose to complete the primary series. Our investigation will strictly confine itself to the impact of mandates on vaccine uptake, intentionally excluding the evaluation of mandates' effectiveness on disease mitigation, mortality, hospitalisation rates or broader economic outcomes. We will construct our outcome variable by considering two types of data:

- Time series aggregated data: for this, our outcome variable will be the total cumulative number of vaccine doses administered over time on a weekly or daily basis per 100 000 people; the percentage of people from the total population with at least one, two, three, four, five or more vaccine doses over time on a daily/weekly basis. This time series data will be constructed at national, state, local government administrative area and postcode levels (when possible) and within subgroups of interest (ie, healthcare workers).
- Individual-level linked data: this refers to COVID-19 vaccination status of an individual at a point in time: If a person ever had a COVID-19 vaccine? and the number of COVID-19 vaccine doses taken? For this, our outcome will be either in a binary form (having COVID-19 vaccine or not (0/1)), or in ordered form (having 0, 1, 2, 3, 4, 5 and above COVID-19 vaccine doses) or count form (the number of COVID-19 vaccine doses taken).

##### Australia

For each endpoint, we will construct vaccine uptake by setting all those with a COVID-19 vaccination record in the AIR to a value of 1 and those that do not have a COVID-19 vaccination record to a value of 0. Furthermore, we will construct our outcome variable by considering COVID-19 vaccination status of an individual at a point in time: if a person ever had COVID-19 vaccine and the number of COVID-19 vaccine doses received. For this, our outcome will be either in a binary form (having COVID-19 vaccine or not (0/1)), or in ordered form (having 0, 1, 2, 3, 4, 5 or more COVID-19 vaccine doses) or count form (the number of COVID-19 vaccine doses received).

##### USA (California)

We will estimate the proportion of the population with at least one, two, three, four, five and more vaccine doses over time on a weekly basis and will use state-level weekly/daily proportion of population with at least one, two, three, four, and more vaccine doses as our primary outcome variable of interest.

##### Italy and France

We will use country-level daily/weekly proportion of population with at least one, two, three, four and more vaccine doses as our outcome variable of interest.

### Policy intervention

We will provide/delineate the chronology/timing of each vaccine mandate, detailing the dates of announcement, implementation and removals, along with the specific rules accompanying each phase and the policy target (the specific population group to whom the mandate applies, if relevant). A jurisdiction/state/country is recognised/considered as implementing employment or public space mandates if workers across different settings are required to provide evidence of vaccination to work in their usual workplace or if the general public is required to provide evidence of vaccination to access public spaces/communal venues such as restaurants or social events. Then, using announcement and implementation dates in each jurisdiction and country (as relevant), we will construct a policy variable indicating pre-mandate and post-mandate changes.

We will also consider the possibility of dual implementation dates, when relevant: the initial enforcement onset of the mandate (*primary analysis*) and the subsequent date when penalties for non-compliance (*secondary analysis*), such as the inability to work, were applied. For instance, in Western Australia, healthcare workers experienced a gap (1–2 months) between the introduction of the mandate-when proof of vaccination was required-and the implementation of repercussions (action) for non-compliance (eg, inability to work if they were not vaccinated).

### Controls/covariates

To the extent possible, we will control for contextual factors affecting COVID-19 vaccine uptake, including the number of local infections/deaths, socioeconomic and demographic characteristics (ie, population density by age group-age is a key given the way COVID-19 vaccines were rolled out), education status, family identifiers including the number of children, household size, income per capita or household income, per cent of population from culturally and linguistically diverse backgrounds), external factors (border closures/lockdowns), regional-fixed and time-fixed effects and the presence of competing control measures (ie, other types of vaccine mandates). When possible, we will control for distance to healthcare as a measure of access. We will also use SEIFA, remoteness index, citizenship status, country of birth, year of arrival in Australia, religious affiliations and other rich set of socioeconomic variables in the census, where feasible/possible. A comprehensive overview of the proposed outcome variables and covariates is presented in online supplemental table A1.

### Study population

Our study population in Australia will include those with a Census 2021 record linked to the PLIDA Spine, without a linked death registration record, who were considered Australian in January 2021. We will exclude people who had missing information on age or sex on the PLIDA Spine. For the overseas comparators, we will construct national or regional trend data for the USA (California), France and Italy with information on daily/weekly COVID-19 vaccine administration.

### Data access for population-based individual linked data

The AIR will be the main source of data for vaccination records in Australia. Linked PLIDA will be accessed through the ABS’ secure DataLab.^13^ Access to the AIR-PLIDA linked data has been formally approved by the ABS. This will be used for in-depth analysis using a range of statistical software packages. The AIR-PLIDA project links frequent updates from the AIR with a range of other Australian Government datasets. This allows us to analyse vaccination coverage among different priority or vulnerable population groups.

### Data access for time series data

For the Australian (national level), the USA (California), Italian and French studies, Our World in Data will be the main source of data for vaccination records. Detailed statistics on vaccine administration, testing, hospitalisations and deaths-broken down by county and further stratified by race, ethnicity and age groups-from the California Department of Public Health’s website^9^ will be used. In addition, vaccination data from the ECDC and TESSy will be used for Italy and France. We will also use data from the Italian Ministry of Health and SPF to get detailed vaccination records by age groups (when appropriate). All these data sources are publicly available. ECDC requires data use in line with ECDC’s copyright policy: ‘Information and documents made available on ECDC web pages and for which ECDC owns the copyright are public and may be reproduced, adapted and/or distributed, totally or in part, irrespective of the means and/or the formats used, provided that ECDC is always acknowledged as the original source of the material. Such acknowledgement must be included in each copy of the material. Citations may be made from such material without prior permission, provided the source is always acknowledged. The copyright policy of ECDC is compatible with CC BY 4.0 licence.’

### Data analysis

We will provide descriptive statistics at the national, state and local statistical areas as well as in the subpopulation groups classified by sociodemographic and employment characteristics. Similarly, we will use descriptive statistics such as averages, SD and minimum and maximum values for each variable that will be included in our regression analysis. Furthermore, we will generate graphs to visually represent the patterns of vaccinations and other relevant variables. The graphs will include time series plots of vaccinations and other contextual factors, such as COVID-19 cases and deaths, as well as event-study plots using the estimated coefficients.

#### Individual linked data analysis

We will use advanced causal inference methods such as difference-in-differences (DID) and synthetic control DID^14 15^ to obtain the estimated effects of vaccination policies on vaccine uptake. We will develop our model to make vaccination uptake dependent on policy variables (mandate and removals), controlling for relevant covariates. To capture different policy responses in different settings, interaction terms will be included in the models. Analyses will be replicated for each jurisdiction/state, allowing for replication and sensitivity testing in different settings, populations and employment groups when the inclusion of the interaction term is not applicable.

As individual vaccination uptake is a binary outcome, we will apply the logit/probit regression models. We will calculate the number of COVID-19 vaccine doses taken by individuals at an observed time to use ordered/multinominal logit/probit or Poisson regression models. In particular, we will apply the DID model, which is specified as follows:


$$Y_{ist}=\;\alpha +\beta_{1}Mandate_{ist}+X_{t}\gamma +\lambda_{i}+g_{s}+\delta_{t}+\epsilon_{ist},$$

where:

- $Y_{ist}$ is the outcome variable (vaccine uptake) for individual i, state s and at time t.
- $Mandate_{ist}$ is a binary variable indicating the presence of a vaccine mandate.
- $X_{ist}$ is a vector of control variables.
- ${\lambda_{i},g_{s}and\delta }_{t}$ represent individual, country/state and time fixed effects, respectively.
- $\epsilon_{ist}$ is the error term.

However, a recent methodological literature has argued that the standard ordinary least squares two-way fixed effects (TWFE) estimator can be invalid in panel data settings with staggered adoption and heterogeneity in the mandate effect across cohorts or states (In this literature, ‘cohort’ is defined as the calendar period in which the treatment starts. So, for instance, all units that start treatment in May 2021 are a cohort. "If treatment effects differ across cohorts, one can either extend the TWFE approach by interacting the treatment variable with cohort-specific dummies, or alternatively, use a different estimator such as Kellogg *et al*^15^) and/or over time. We will resort to models that would consider the staggered and heterogeneous nature of the mandate implementation.

To be more specific, Callaway and Sant’Anna^16^ separate the analysis into identification and estimation/inference that allows the use of additional covariates, different comparison groups, panel and repeated cross sections. They first estimated the ATT(g,t)-the average treatment effect at time t of starting treatment (mandate implementation) at time g. The ATT(g,t) can be estimated using either the ‘never treated’- those states that have never implemented the mandate-or the ‘not yet treated’- those states that have not yet implemented the mandate-as comparison groups. This means the problem is broken down into many 2×2 DIDs, using only good designs. After identifying all feasible $ATT(g,t{)}^{\prime }s$, they came up with a series of aggregations to have one ATT.

As a robustness check and if data allow, that is, if we have a balanced panel, we will also use the *synthetic* DID by Arkhangelsky *et al*^14^ that combines the synthetic control method with the DID approach by constructing a synthetic control group using the same approach as in the synthetic control method. However, the treatment effect is estimated by comparing the change in outcomes between the treated unit (those that implemented the mandate) and the synthetic control group before and after the treatment is introduced. To construct suitable control jurisdictions, we will select comparator regions that exhibit similar preintervention trends in vaccine uptake and closely aligned demographic characteristics with the treated jurisdictions. This approach aims to ensure comparability and strengthen the validity of the estimated policy effects.

Any missing personal/demographic data will be reported using descriptive statistics. Individuals with some missing data for key demographic variables will be excluded. We will check if data is missing at random and impute missing values when necessary. We will use multiple imputation or complete case analysis, depending on the nature and extent of missingness.

All analyses of AIR-PLIDA data will be conducted within the DataLab, a secure government environment specifically designed to safeguard sensitive information. DataLab provides a platform for end users to conduct statistical analysis of detailed microdata within a secure environment. Access to DataLab is contingent on completion of mandatory Safe Researcher Training, which familiarises users with their legislative responsibilities and the protocols for data output clearance by the ABS. This training is essential for new users to gain approval to work on projects within DataLab, and the training focuses on compliance and ethical use of data. The registration process for training is streamlined through the myDATA portal, where users can manage their training, onboarding and project participation. Researchers must also complete periodic refresher training and recommit to DataLab’s declarations every 2 years to maintain their access. DataLab itself is designed to ensure the security of microdata analysis through robust features such as encrypted data storage, onshore cloud servers and multifactor authentication. All outputs intended for external use undergo rigorous checks by the ABS to prevent unauthorised data disclosure. DataLab is hosted on Microsoft Azure, ensuring compliance with the highest governmental security standards (PROTECTED level - the 'PROTECTED' classification refers to sensitive information that, if disclosed without authorization, could cause damage to national interests, organizations, or individuals), further safeguarded by ongoing security audits and threat detection mechanisms. The platform supports various statistical software, including R, SAS, Stata and Python and ensures all analytical outputs are vetted before release. DataLab’s structure adheres to the safe settings principle, part of the Five Safes Framework, ensuring that data privacy and security are paramount in every aspect of its operation.

#### Aggregate data analysis

#### Interrupted time series analysis (ITSA)

Here, we will assess changes in vaccine uptake trends before and after mandate announcement and removal. We will analyse weekly data, and (when possible) we will consider different time windows before and after mandate implementation and removal. The ITSA model specified below includes autoregressive terms to account for serial correlation and seasonal components, which can be done by including lagged values of the outcome variable and seasonal dummy variables in the model.^17^,^19^


$$Y_{t}=\text{\textbackslash{}boldsymbol}\;\alpha +\beta_{1}T_{t}+\beta_{2}Mndate_{t}+\beta_{3}\left(Mndate_{t}*T_{t}\right)+\text{\textbackslash{}boldsymbol}X_{\text{\textbackslash{}boldsymbol}t}\gamma +\epsilon_{t},$$

where Yt represents the outcome variable at time t, Tt represents the time since the start of the study (often measured in time units before and after the intervention), ${Mndate}_{t}$ is a dummy variable representing the intervention—that is, the mandate announcement (preannouncement period 0, otherwise 1) and ${Mndate}_{t}*T_{t}$ is an interaction term. The coefficients $\beta_{2}$ captures the immediate effect of the mandate announcement and removal, $\beta_{3}$ captures the change in slope after the intervention. If autocorrelation is detected, appropriate lags need to be included.

As a robustness check, we will consider using the Regression Discontinuity in Time (RDiT). The RDiT is a regression discontinuity with time being considered as a running variable. We define the cut-off point as the point in time when the vaccine mandate was announced and implemented. The policy variable is, thus, defined as a binary indicator taking a value of 1 for the period after the mandate implementation and 0 otherwise.^20^

### Ensuring impact

The broader MandEval research project, in which this study sits, has established a community advisory group to advise on the development of the project and dissemination and to maximise the policy contribution of this research. The advisory group consists of people with diverse expertise and perspectives from across Australia. This group will be consulted throughout the project. Virtual sessions will be organised when developing the project to ensure policy relevance and to discuss our findings with the aim of translating the findings into messages for policy. Moreover, findings will be shared with Commonwealth and state governments through investigators and established relationships with formal government project partners across Australian states, as well as the broader community through newsletters, publications, conference presentations and forums.

### Patient and public involvement

In addition to the advisory group described above, we have formal consumer representation on our governance group. This representative will advise on the development of the project and reporting of results.

## Ethics, dissemination and translation

A favourable ethical approval was obtained from the human research ethics committee (HREC) at the University of Newcastle with a *protocol number: H-2024-0160*. Amendments to the protocol or other study documents will not be implemented without these approvals. If there is a need to deviate from the protocol, the nature of and reasons for the deviation will be documented and submitted to the HREC. If this necessitates a subsequent protocol amendment, this will be submitted to the HREC for review and approval.

Results will be disseminated via briefings, newsletters, webinars to the public health community and government policy-makers (informed by our governance group) and to the academic community (via journals and conference presentations). Project information will be reported on the publicly available MandEval website (pending construction), and we will use Vhep’s Blog and social media accounts to disseminate key findings. Findings will be presented at national/international conferences and peer-reviewed journals.

Ownership of the data results arising from this study resides with the study team and their employer and the study’s funder. On completion of the study, a study report will be prepared. The study report will be used for publication, teaching purposes and presentation at scientific meetings. At the commencement of the project, a core team will engage in an initial authorship discussion to determine the eligibility for authorship, focusing on various aspects such as contributions to the research design, execution or manuscript writing. This stage includes a nomination process where the core team collectively decides on individual roles and contributions. Throughout the project, a detailed record of contributions will be maintained to ensure that substantial contributions result in authorship while minor assistance is recognised separately. The use of the Contributor Roles Taxonomy (CRediT) taxonomy (The CRediT is a standardised system used to describe the specific contributions of individuals involved in scholarly research^21^) will aid in maintaining transparency regarding the contributions of each author. The strategy for determining authorship will prioritise inclusivity, mandating an open dialogue within the team concerning potential authors. When it comes to external collaborators, any involvement must be prediscussed with the core team. Additional criteria for authorship may be established by the core team as necessary. Investigators have the right to publish orally or in writing the results of the study. Summaries of results will also be made available to investigators for dissemination within their specialty areas (where appropriate and according to their discretion).

In addition to publishing and providing policy guidance, the project’s final year synthesis combines the findings into high-level advice for policymakers, including efforts to ensure our recommendations are reflected in future pandemic planning documents.

## Supplementary material

10.1136/bmjopen-2024-097412 online supplemental file 1

## Data Availability

Data for individual-level analysis will be sourced from AIR-PLIDA, with access and sharing restricted to authorized parties. However, guidance and code for accessing the data will be provided. Aggregate-level analysis will use publicly available datasets, which will be fully accessible.
