# Supplementary material for: Impact of vaccine mandates and removals on COVID-19 vaccine uptake in Australia and international comparators: a study protocol
Source: BMJ Open. 2025 Jul 7;15(7):e097412. doi: 10.1136/bmjopen-2024-097412 (PMC12258311; doi:10.1136/bmjopen-2024-097412)
Supplement: online supplemental file 1 [file bmjopen-15-7-s001.docx]

Table A1: Variables across jurisdictions

| Category | Variable | Australia (AIR-PLIDA) | California (USA) | France | Italy |
| --- | --- | --- | --- | --- | --- |
| Vaccination outcome measures | Any dose received (binary) | Individual-level | Aggregated | Aggregated | Aggregated |
|  | Number of doses received (count) | Individual-level | Aggregated | Aggregated | Aggregated |
|  | Booster dose received | Individual-level | Aggregated | Aggregated | Aggregated |
|  | Weekly vaccine uptake per 100k | Aggregated or derived | Aggregated | Aggregated | Aggregated |
| Sociodemographic variables | Age group | Individual-level (Census) |  |  |  |
|  | Sex | Individual-level (Census) |  |  |  |
|  | Occupation (e.g. healthcare/education) | From Census and tax records |  |  |  |
| Policy timing variables | Mandate announcement/removal date | Available | Available | Available | Available |
| Contextual factors | COVID-19 case/death trends | Available (state-level) | Available | Available | Available |
