# Supplementary material for: Impact of vaccine mandates and removals on COVID-19 vaccine uptake in Australia and international comparators: a study protocol
Source: BMJ Open. 2025 Jul 7;15(7):e097412. doi: 10.1136/bmjopen-2024-097412 (PMC12258311; doi:10.1136/bmjopen-2024-097412)
Supplement: Supplementary file 2 [file bmjopen-2024-097412.reviewer_comments.pdf]

PEER REVIEW HISTORY

BMJ Open publishes all reviews undertaken for accepted manuscripts. Reviewers are asked to complete a checklist review form and are provided with free text boxes to elaborate on their assessment. These free text comments are reproduced below.

ARTICLE DETAILS

Title (Provisional)

Impact of Vaccine Mandates and Removals on COVID-19 Vaccine Uptake in Australia and International Comparators: A Study Protocol

Authors

Gebremariam, Aregawi Gebremedhin; Genie, Mesfin; Le, Huong; Attwell, Katie; Liu, Bette; Regan, Annette K; Beard, Frank H.; Macartney, Kristine; Paolucci, Francesco; Moore, Hannah Catherine; Blyth, Christopher C.

VERSION 1 - REVIEW

**Reviewer**	**1**
**Name**	**Yuan, Meng**
**Affiliation**	**Chongqing University**
**Date**	**09-Mar-2025**
**COI**	**None**

The manuscript has clearly elaborated their research questions and methods.

**Reviewer**	**2**
**Name**	**Reddon, Hudson**
**Affiliation**	**UBC**
**Date**	**09-May-2025**
**COI**	**None**

Thank you for the opportunity to review this protocol. The authors propose to evaluate and quantify the impact of COVID-19 vaccine mandates and removals on vaccine uptake in multiple countries.

The protocol includes analyses of data from Australia, Europe and the US to investigate these effects in different jurisdictions. This work is an important contribution to pandemic preparedness and vaccine mandates, and is particularly timely given vaccine hesitancy with other diseases such as measles. My specific comments are included below.

1. Abstract

Minor comments

-None.

2. Introduction

Minor comments

-Table 1. Adding the number of participants in the population column, and the percent increase in vaccination rates in the findings column would be informative for the reader and demonstrate the effect size of these interventions.

-Aims. Will the outcome include any dose, completion of vaccine protocols or boosters? If the mandates differed by country this would be important to include.

-Page 11. Line 14. I don't think it is accurate to say that the study will deliver an evidence base as this is just one study. I'd suggest rewording.

3. Methods

-Australian datasets are described in detail.

-A table outlining what variables can be harmonized across datasets would be helpful to evaluate the potential to pool data and the subgroups and regional factors that can be compared.

-Page 17. Line 37. What kind of graphs will be generated? More detail about what measures will be analyzed here would be helpful.

-Page 17. Line 50. Causal impacts of vaccination cannot be obtained from theses methods and can only be inferred. Suggest refraining from "causal" language particularly when using population level data. More appropriate to use associations to describe these effects.

-Page 18. Line 45. OLS should be spelled out after first use.

-Page 19. Line 30. What settings or regions will be used for the synthetic control? How will you ensure that they are similar to the settings with the mandates?

-Who will be included in the advisory group? Community, researchers, policy makers etc.?

VERSION 1 - AUTHOR RESPONSE

Reviewer 1 – Dr. Meng Yuan

General Comment: "The manuscript has clearly elaborated their research questions and methods."

Response: We thank the reviewer for their positive and encouraging feedback.

Reviewer 2 – Dr. Hudson Reddon

Introduction

1. Comment: “Adding the number of participants in the population column, and the percent increase in vaccination rates in the findings column would be informative.”

Response: Thank you for the informative comments. We have updated the table adding the number of participants and increase in vaccination rates if indicated on pages 5-7. As most of the studies utilized time series data, we have indicated the study start and end dates along with the countries/states.

2. Comment: “Aims. Will the outcome include any dose, completion of vaccine protocols or boosters? If the mandates differed by country this would be important to include.”

Response: We have clarified in the Aims sections (page 8, paragraph 1, line 33) that the primary outcome is the uptake of any vaccine dose, with secondary outcomes including completion of the primary vaccination series and receipt of booster doses, where data allow.

3. Comment: “Page 11. Line 14. I don’t think it is accurate to say that the study will deliver an evidence base as this is just one study. I’d suggest rewording.”

Response: We have reworded this phrase to: “...to contribute to the evidence base on the effects of vaccine mandates and their removals...”. Refer page 9, paragraph 1.

Methods

4. Comment: “Australian datasets are described in detail. A table outlining what variables can be harmonized across datasets would be helpful to evaluate the potential to pool data and the subgroups and regional factors that can be compared.”

Response: We thank the reviewer for this helpful suggestion. We agree that outlining the potential for data harmonization across countries and jurisdictions is critical for understanding the comparability and potential for pooled or parallel analyses. To address this, we have now added a supplementary table in the appendix (Table A1) that outlines key variables across the Australian, US (California), French, and Italian datasets. We have added a note at the end of paragraph 2 on page 16 as follows “A comprehensive overview of the proposed outcome variables and covariates is presented in Table A1”.

5. Comment: “Page 17. Line 37. What kind of graphs will be generated? More detail about what measures will be analyzed here would be helpful.”

Response: We have updated the relevant sentence to clarify that the graphs will include time series trend plots of vaccinations and COVID 19 cases/deaths, and Event-study plots. Refer page 18, end of paragraph 2.

6. Comment: "Page 17. Line 50. Causal impacts of vaccination cannot be obtained from theses methods and can only be inferred. Suggest refraining from "causal" language particularly when using population level data. More appropriate to use associations to describe these effects."

Response: Acknowledging the limitations of observational data, we have revised all relevant sections to replace references to "causal impacts" with "estimated effects". Refer page 19, paragraph 1, line 2; and the 'Methods and analysis' part of the abstracts.

7. Comment: "Page 18. Line 45. OLS should be spelled out after first use."

Response: We now spell out "Ordinary Least Squares (OLS)" at its first mention in the manuscript. Refer page 20, paragraph 1.

8. Comment: "Page 19. Line 30. What settings or regions will be used for the synthetic control? How will you ensure that they are similar to the settings with the mandates?"

Response: We have clarified that control jurisdictions in synthetic control methods will be selected based on pre-intervention trends and demographic characteristics to closely match treated jurisdictions. Refer page 21, paragraph 1.

9. Comment: "Who will be included in the advisory group? Community, researchers, policy makers etc.?"

Response: We have updated the manuscript (page 23, paragraph 3) to clarify the composition of the advisory group that includes individuals with diverse expertise and perspectives.

We hope that our revisions satisfactorily address all reviewer and editor(s) comments. We appreciate the opportunity to improve the manuscript and look forward to your further feedback.
